# Aligners as a Therapeutic Approach in Impacted Canine Treatment: A Systematic Review

**DOI:** 10.3390/jcm14103421

**Published:** 2025-05-14

**Authors:** Mateusz Wolny, Agata Sikora, Aneta Olszewska, Jacek Matys, Agata Czajka-Jakubowska

**Affiliations:** 1 Department of Orthodontics and Temporomandibular Disorders, Poznan University of Medical Sciences, 61-701 Poznan, Poland; mz.wolny2@gmail.com (M.W.); anetaol@ump.edu.pl (A.O.); a.czajka-jakubowska@ump.edu.pl (A.C.-J.); 2 Faculty of Medicine, Poznan University of Medical Sciences, 61-701 Poznan, Poland; 83521@student.ump.edu.pl; 3 Department of Dental Surgery, Wroclaw Medical University, 50-425 Wroclaw, Poland

**Keywords:** orthodontic aligners, impacted canine, ectopic canine, impacted tooth, ectopic tooth

## Abstract

**Background/Objectives:** The growing demand for esthetic, less painful, and more comfortable orthodontic treatment has led to increasing use of aligner systems. Initially used for less complicated malocclusions, aligners are now being incorporated into complex treatment plans, including cases involving impacted teeth. While aligners are a popular alternative to traditional fixed appliances, they still have limitations. This study aims to evaluate the effectiveness of aligner-based orthodontic treatment in patients with impacted or significantly ectopic canines. **Methods:** This study was conducted in accordance with the PRISMA guidelines. The search terms used were as follows: ‘Clear Aligner’ OR ‘Invisalign’ AND ‘Impacted Canine’ OR ‘Impacted Tooth’ OR ‘Ectopic Tooth’ OR ‘Ectopic Canine.’ A total of 1101 records were identified, of which 170 articles underwent screening. Fifteen articles were assessed for eligibility, and ultimately six case reports and one three-dimensional finite element analysis (FEA) study were included for both quantitative and qualitative synthesis. **Results:** According to the studies, additional appliances are often required to achieve favorable outcomes when treating impacted canines with aligner systems. Temporary anchorage devices (TADs) were used in 5 out of 9 reported cases for canine traction into the dental arch. In three cases, TADs were combined with sectional wires implemented as cantilevers. Elastics were used in 6 out of 9 cases for traction to the opposite arch, and in 5 out of 9 cases as interarch elastics attached to the aligners. Interarch elastics were applied in various ways, either directly to the aligners or to primary canines using hidden buttons inside pontics or dovetail hooks. Elastics were also anchored to the lower arch with class II, class III, or cross-arch (criss-cross) mechanics. **Conclusions:** This review highlights the promising potential of aligner systems in the treatment of impacted canines. However, additional auxiliaries, such as TADs, sectional wires, or elastics remain nearly essential for initial canine traction. Aligner systems offer versatile treatment options, and the possibility of reduced treatment time represents a valuable area for future research.

## 1. Introduction

Clear aligner orthodontic treatment is becoming increasingly popular among patients, solving some disadvantages of fixed appliances with appealing aesthetics, comfort, and better oral hygiene. When aligner systems were first introduced, they were far from perfect, presenting issues with torque control, rotations, and tooth translation [1]. however, more complex cases can be solved successfully due to the rise in clinical knowledge and technical improvements made by orthodontic companies [2]. Nowadays, standard protocols implemented into aligner treatment can expand the arches, correct crossbites, distalize teeth, and correct deep bites with much more precision and ease compared to analogical methods from just 5 years ago. With aligners’ evolution, the biomechanics behind the treatment have changed from force-driven tooth-by-tooth movement to more complex (but also usually more effective) aligner-driven movement, with the whole arch operating altogether to achieve the final tooth position. Still, some malocclusions are a big concern, but with patients demanding more, especially adults, there is a need for research on alternative treatment options to standard fixed appliances to meet their expectations. Full aligner treatment and the hybrid approach (with orthodontic temporary anchorage devices (TADs) or sectional fixed appliances) are promising treatment options.

One of the ongoing orthodontic problems in the human population is ectopic or even impacted canines. According to a study [3], the incidence of canine inclusion ranges from 1% to even 5.9%. Although impaction can occur in various locations, including the upper and lower jaws and both sides of the alveolar ridge, the most common site is the palatal side of the maxilla [4]. The second most frequent location is also in the maxilla, with the canine positioned buccally [4]. Impaction can be caused by several internal and external factors, including a lack of space for eruption in the arch [5], an abnormal eruption path [4], disposition of the tooth bud [3], supernumerary teeth [4], and even genetic factors [3]. Persistent primary dentition is one of the most common reasons for the lack of or ectopic canine eruption [3]. This problem needs to be solved as soon as it is observed. Otherwise, it can lead to other disturbances in the adjacent teeth. Positive deflection of the crown of the lateral incisor [5], resorption of the adjacent tooth roots [6], or their increased mobility [7], and finally, even tooth loss are among possible consequences [8,9].

Several orthodontic techniques have been proposed and are used to treat impacted canine malocclusion, including surgical approaches and different combinations of specific devices, such as trans-palatal arches, implant-supported disimpactor systems, and the BENEfit distalslider. The typical approach mainly consists of creating a space in the dental arch for the missing canine with fixed orthodontics [10], followed by the surgical exposures of the canine to promote eruption [11]. To reduce the size of appliances necessary for the treatment for patient’s comfort improvement, many sectional wires are frequently implemented into the treatment. The Kilroy spring system and nickel–titanium closed-coil spring successfully relocate impacted canines [12]. The cantilever system is also used predictably in these cases [10]. In some very complicated cases, the best solution could be to extract the canine [13]. However, if it is possible, canines should be aligned into the arch due to their important esthetic and occlusal values. For this reason, there is ongoing research and development of impacted canine treatment methods. One of the new approaches for maxillary impacted canines is the “canine first technique”, with surgical exposure as the first step of the treatment [14]. Additionally, TADs have been widely and successfully used as anchorage devices to overcome some limitations of fixed appliances and aligners [15,16]. With all the success of modern techniques for impacted canine treatment, there is a rising opportunity to merge these techniques with the aligner treatment.

With the development of orthodontic aligner treatments and the increase in patients’ demands, new possibilities for the existing problems must be sought. This study aims to research the effectiveness of aligner orthodontic treatments in treating patients with impacted or ectopic canine. There has been no previous systematic review conducted regarding this subject.

## 2. Materials and Methods

### 2.1. Protocol

**Figure 1:**
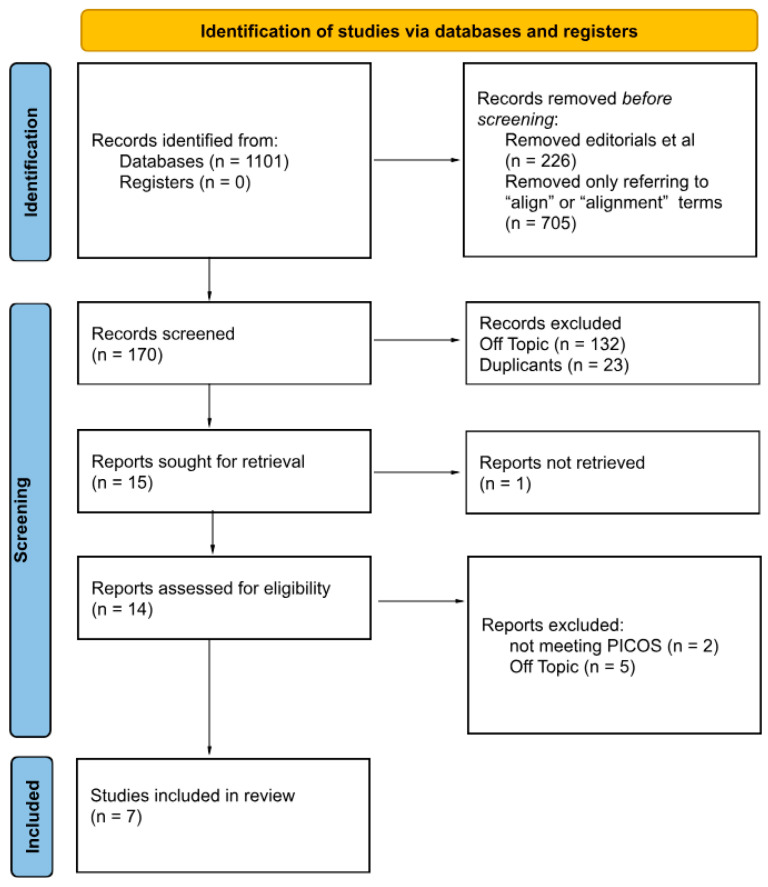
The PRISMA 2020 flow diagram.

This study was conducted in strict accordance with the PRISMA (Preferred Reporting Items for Systematic Reviews and Meta-Analyses) guidelines [17]. The systematic review was registered in the Open Science Framework at the following link: https://doi.org/10.17605/OSF.IO/AGV74 (Figure 1).

### 2.2. Focus Question and Eligibility Criteria

The research question for this study was “Is it possible to treat cases with impacted or highly ectopic canines with orthodontic clear aligners?”. The inclusion and exclusion criteria followed the PICOS (Population, Intervention, Comparison, Outcomes, Study Design) framework [18] and are listed in Table 1. The criteria for inclusion for the review covered orthodontic treatments carried out using an orthodontic aligner system before or after impacted canine exposure and traction into the arch. Different auxiliaries were included in this study, including the use of fixed appliances before treatment with aligners. There were no limitations regarding geographical scope or publication time frame. Reviews, editorials, commentaries, conference abstracts, and books were excluded. Studies with only fixed appliance treatment or those that did not include aligner orthodontic treatment were not considered. Only articles that were published in English and with available full-text versions were included. Articles that failed to match the PICOS framework were also excluded from the review (Table 1).

### 2.3. Search Strategy, Information Sources, and Study Selection

Data were collected until September 2024 with a meticulous electronic research of scholarly databases. Multiple combinations of search terms were used to extend the search range and cover all possible relevant studies in different databases. The searched index terms were ‘Clear Aligner’ OR ‘Invisalign’ AND ‘Impacted Canine’ OR ‘Impacted Tooth’ OR ‘Ectopic Tooth’ OR ‘Ectopic Canine’. ‘SPARK orthodontic system’ as a term word was excluded from the search due to inadequate search results from the databases. Various databases were included in the process, including PubMed, Google Scholar, EBSCO, Web Of Science, Complementary Index, MEDLINE Ultimate, Springer Nature Journals, Oxford Medicine Online, Academic Search Ultimate, Dentistry and Oral Sciences Source, and Elsevier. The strategy was to allow linked words and terms to be searched manually, covering the full text of the articles. Nevertheless, the Preferred Reporting Items for Systematic Review and Meta-analysis (PRISMA) [17] guidelines were carefully followed. The search criteria are thoroughly described in Table 2. The reference lists of the included articles were also checked for any additional relevant studies. Supplementary searches were conducted in journals pertinent to oral surgery and orthodontics. Due to the lack of randomized trial studies, case reports were included. These circumstances may cause bias due to the absence of control study groups (Table 2).

### 2.4. Data Collection Process and Data Items

Two reviewers (M.W. and A.O.) independently performed data extraction from articles that satisfied the predefined inclusion criteria. The gathered information was precisely recorded in a standardized spreadsheet for systematic organization and analysis. The variables assessed were study ID, study design, age, gender, malposition of the canine, TADs, elastics usage, fixed orthodontic appliance usage, period, and number of orthodontic aligners used per treatment.

### 2.5. Risk of Bias and Quality Assessment

To reduce potential bias, in the first phase of study selection, each reviewer performed independent revision of the titles and abstracts. Furthermore, to evaluate the level of agreement among the reviewers, Cohen’s kappa test was used. Any disagreements about including or excluding an article were resolved through discussions among the authors according to the guidelines [19].

### 2.6. Quality Assessment

Two independent assessors (M.W. and A.O.) systematically evaluated the methodological rigor of each study to decide on its inclusion. In the event of a disagreement between the reviewers regarding the inclusion of an article, a third reviewer was consulted to resolve the dispute and make the final decision. For the quality assessment, a set of critical appraisal tools developed by the Joanna Briggs Institute (JBI) was used [20].

Cohen’s kappa test was performed to assess the inter-rater reliability using MedCalc version 23.1.7 (MedCalc Software Ltd., Oostende, Belgium). The test results show a kappa value of 0.92, indicating almost perfect agreement and high consistency among the reviewers’ assessments.

## 3. Results

### 3.1. Literature Search Results and Study Selection

The first round of research was conducted in various databases and registers, and 1101 records were obtained during the initial identification. After removing the excluded articles, including editorials, books, and other non-research articles, as well as articles only referring to the term “align” or “alignment”, 170 articles were included in the screening process. After the second removal of residual articles that did not meet the inclusion criteria and duplicates, 15 articles were selected for retrieval. No clinical studies were found at this point in the research, so case reports were included. One report could not be completely accessed, five were off-topic, and two articles failed to meet the PICOS criteria. After these exclusions, six case reports [21,22,23,24,25,26] and one three-dimensional finite element analysis (FEA) study [27] were eligible for this review for both quantitative and qualitative synthesis.

Qi Fan et al.’s study was excluded from the research due to its focus on the traction of an impacted lower third molar. M Palone et al. [28] presented a case with an impacted lateral incisor, not an impacted canine. Most cases were female (6/9) [21,22,23,24,25], with most originating from Italy (8/9) [21,22,23,24,25,26], and the median age was 17; the oldest was 43 [21], and the two youngest were 13 [21,25]. Most studies consistently reported that impacted canines occurred more frequently in female compared to male patients, indicating that this malalignment applies to the maxilla rather than the mandible [29]. Qijan Xia et al.’s study [27], a three-dimensional finite element analysis (FEA), created four 3D biomechanics models of a Chinese male patient and investigated two possible clinical scenarios. The remaining articles were case studies due to the lack of trial or comparative studies.

Due to the low number of case studies, the lack of control groups, and no sample sizes bigger than a singular patient, there is a high risk of bias. The patients were in a relatively young group (15–30 years old) and were predominantly female. Nevertheless, there was a difference in the impacted canine positions.

### 3.2. Study Characteristics

General and detailed characteristics of the incorporated articles are presented in Table 3 and Table 4. The Invisalign@ [21,22,23,24,25] system was most commonly used across the studies. Qijan Xia et al.’s FEA study [27] used a Young’s modulus of 816.31 MPa and a Poisson’s ratio of 0.3 for their calculations. Bochinno et al. [26] used UAB Ordoline (Vilnius, Lithuania) aligners. No studies using the SPARK@ (ORMCO, Orange, CA, USA), Angelaligner@ (Angelalign Technology Inc., Shanghai, China), or other systems were conducted. Two cases were presented with highly ectopic canines, one positioned buccally [21] and one palatally [25]. The rest were impacted primarily on the palate, corresponding with occurrences in the general population. Most of the included patients chose aligner orthodontic treatment due to aesthetic reasons after considering the different treatment mechanisms. Various treatment plans were implemented. Canine tractions were conducted using elastics or cantilever springs with TADs, and, in some cases, even both appliances. Two main strategies were used—the canine-first approach and space creation in the dental arch for tractioned teeth. With palatally impacted canines, bite disocclusion on the molars or premolars was planned. Every case presented in this article resulted in aligning the canines into the arch and correcting malocclusion (Table 3).

### 3.3. Main Study Outcomes

During the analysis of the included articles, several consistent trends and treatment strategies emerged. It is possible to treat impacted or ectopic canines using clear aligner systems, but the majority of cases required additional auxiliaries to achieve predictable results. TADs were used in 5 out of 9 cases [22,24,26], primarily for canine traction into the dental arch. In three cases, they were combined with sectional wires in a cantilever configuration [22,24,26]. Elastics were also widely applied. They were used during traction in 6 out of 9 cases for opposite arch anchorage [22,24,25,26,27], and in 5 cases as interarch mechanics attached to the aligners [21,23,24,26,27]. These elastics were applied in various configurations, either directly to the aligners, to the primary canines, or with hidden attachments, such as buttons inside pontics [23] and dovetail hooks [23]. The elastics were anchored in different classes (II [21,24,25], III [26], or criss-cross [22]), showing versatility in vector control.

A variety of attachments were implemented to support traction. An FEA study [27] showed superiority of the power arm and orthodontic buttons over a 3D-printed attachment, with the power arm being more appropriate when the impacted canine was positioned above the lateral incisor. The most used technique was an orthodontic button with elastics attached [21,22,23,24,25,26,27], and one case used hooks on the cuspids [23]. The cases presented also noted the importance of bite disocclusion during the traction of palatally positioned canines to remove any interferences and minimize tooth collisions [22,24,25,26].

Most studies applied a three-phase treatment strategy, consistently dividing the process as follows: (1) space creation and arch development, (2) surgical exposure and traction (often with auxiliaries), and (3) final tooth alignment and torque correction [21,22,23,24,25]. Variants included canine-first approaches [22,24,26], initial arch expansion [21,23,25,27], or simultaneous space opening and traction in later phases [24]. Every case presented used aligners during the final stage, mainly for torque correction and final teeth alignment. Qijan Xia et al.’s study [27] presented minimal differences in the biomechanical effects of impacted canine traction with clear aligner therapy compared to the fixed appliance mode. However, clear aligners without prescribed attachments caused more uncontrollable displacement of anchorage teeth, especially lateral incisors and first premolars, and 8–11 times greater periodontal ligament stress than fixed appliances. According to Qijan Xia et al. [27], the fixed appliance promoted better dentition binding for better traction support than aligner materials, which lack adequate stiffness for sustaining the original shape and promoting strong and stable anchorage. However, this was not mentioned as a problem in any other case presented [21,22,23,24,25,27].

Every treatment was conducted in stages, mainly divided into three phases [21,22,23,24,25]. In most cases, the aligners were changed every 7–10 days, with a slower rate of change at the beginning of the treatment. The number of aligners varied significantly in cases where sectional wires were used, ranging from 24 [26] to approximately 52 aligners [24]. Cases using only aligner orthodontic systems without TADs showed a mean of 64 aligners and treatment lasting almost 2 years. With the combined usage of TADs and aligners, it took approximately 50 aligners to treat. A fixed sectional wire further lowered the number of aligners used [26]. The average treatment lasted 24 months in total or less. This suggests that the use of skeletal anchorage and fixed auxiliaries may reduce the total number of aligners and potentially shorten treatment time.

Most cases, with two exceptions [22,24], described the precise number of aligners used and the duration of treatment. Every case was well documented with radiographic projections (most with orthodontic cephalometric analysis), and malocclusions were presented in detail. Moreover, all studies acknowledged the force used during canine traction, but only three studies [23,25,27] showed the size and force of elastics used during the treatment. Only one study, that is, the FEA study, compared orthodontic aligner systems with fixed appliances [27]. Patients made their treatment choices primarily based on their aesthetic demands (Table 4).

### 3.4. Quality Assessment of the Included Studies

Six case reports were evaluated with the checklist. Table 5 presents the scoring for the evaluated studies. Every case report received a high amount of points, with 7 out of 8 earned [21,22,23,24,25,26]. The risk of bias in the FEA study was not assessed due to the lack of a relevant checklist [27] (Table 5).

## 4. Discussion

Nowadays, aligner orthodontic treatment has several advantages over fixed appliances, including patient comfort, easier maintenance of oral hygiene, and aesthetic value, among others [30]. More importantly, aligner treatment provides precise staging, with every single aligner controlling the amount of tooth movement. Forces implemented on the teeth can be adequate to promote traction more biologically and safely [31]. These factors are crucial with sufficient torque and angulation positioning of the canines [32]. Other advantages of orthodontic aligners include less pain perceived by the patient and less frequent appointments [33]. It is also an appealing alternative for patients suffering from metal allergies [34]. Combining clear aligners with a hybrid approach or TADs drastically reduces the need for nuanced appliances for impacted canine treatment, such as transpalatal expanders, full-arch fixed appliances, and additional appliances for increased anchorage [35]. Modern TADs also reduce the number of surgical procedures needed [36]. Nevertheless, aligners have one huge disadvantage: as removable appliances, they require proper dedication from the patient to work.

When essential treatment conducted by extracting the primary canines does not work [37], the need arises for other solutions. Surgical exposure of the impacted canine is usually mandatory. The open eruption technique is preferred due to the lower risk of ankylosis of the tractioned canine (3.5% compared to a 14.5% risk with the closed technique) [38]. The next step in the treatment could be expanding the arch to make space for the canines [39], a process in which orthodontic aligners excel [40]. In addition, bite turbos for disocclusion, eruption guides, and pontics for space management can could be easily implemented into the aligner treatment. Another problem comes with tracing the impacted canines. Aligners are not the best tool to control the anchorage of adjacent teeth [27,41]. Studies are inconsistent on whether aligners are the most precise points of anchorage for the forces needed for canine traction. Upadhyay et al.’s study [42] showed more spread out stress on the periodontium of the adjacent teeth caused by aligner treatment compared to fixed appliances and also pointed out aligners not being efficient at transferring force to teeth. However, the study of Bilello et al. [42] provided data supporting the accuracy of the clear aligner system, and Yang et al.’s study [43] successfully implemented two degrees of anchorage preparation for maximum stability of the anchorage tooth. In addition to the loss of anchorage [44], another leading cause of treatment failure is tooth resorption; almost 0.9 mm of pathological resorption occurs in the roots of teeth with fixed appliance treatment [45,46]. Yuan Li et al.’s study [47] presented significantly lower severity of apical root resorption in the clear aligner group (0.13 ± 0.47 mm compared to 1.12 ± 1.34 mm using fixed appliances). To overcome these difficulties, many authors have used TADs as additional anchorage to precisely disimpact the canine first [48].

It can be assumed that elastics have limited usefulness, promoting only vertical vectors of traction [49], but authors have presented a broad spectrum of traction directions [21,22,23,24,25,26,27]. Different elastic forces can be implemented, including hammock-style over the aligner for vertical traction, class II or class III elastics for vertical–horizontal traction, or put over the eruption guide on the aligners for vertical movement. Additionally, criss-cross elastics can be used for interarch horizontal–vertical force. Qijan Xia et al. [27] proved a lack of significant difference in the vestibular traction of highly ectopic canines between elastics anchored to the aligner and those anchored to a fixed appliance. In some cases [22,26], surgically exposed canines need precise extrusion and distalization force to omit the roots of the adjacent teeth. Using TADs when the exposure of the canine is low can resolve this problem [49]. Micro-screws incorporated into treatment also promote more precise force applied to the tractioned canines [49,50]. Control over these forces is necessary, because excessive forces of more than 20–26 g, combined with prolonged exposure (more than 6–8 weeks), stimulate the process of pathological resorption [51]. On the other hand, when the canine is positioned vestibularly or in an alveolar crest, elastics could be enough [21,23]. During the final stages of treatment, when palatal canines are close to the dental arch crest, they can be aligned by the pushing motion of the aligners, with precise root control. Moreover, disoccluding the patient allows for more collisionless movement of the canines and can be easily implemented into aligner treatment [25,52]. Depending on the distance of the canine, cuspid traction can be further secured with criss-cross elastics [22,24,25,26], or only attachments can be used [21,22,25,52].

The treatment time was similar when comparing the treatment plans of the presented studies with fixed appliances [53], TADs, aligners, and a hybrid approach (aligners + fixed appliance). According to the literature, there is no significant difference in the time between canine traction using mini-screws and the transpalatal arch [54]. Moreover, Alhamwi et al.’s and Flores-Mir’s studies [55,56] showed no significant difference in treatment time between using fixed appliances and clear aligners. However, several studies have suggested that treatment time can be reduced with aligners, thanks to the possibility of multiple dental corrections occurring simultaneously, especially in young patients [57,58,59].

This systematic review presents an up-to-date research summary of impacted canine treatment with clear aligners and different clinical treatment protocols. Nevertheless, this study has its limitations. This study used case studies due to the lack of clinical trials on the subject. The lack of high-quality randomized or controlled trials lowers the possibility of formulating strong clinical conclusions. The authors put in an effort to compare the effectiveness of clear aligners and the fixed appliance treatments. However, due to the lack of precise studies comparing both methods, only a theoretical comparison could be conducted. The cases presented used only a small margin of orthodontic aligner systems available on the market and concentrated on narrow age groups. The authors of the incorporated studies carefully selected the patients presented in the case studies. Moreover, despite wide and meticulous research, not every possible search engine and database was included in this study, leading to the possibility of human error occurring during research. Future research should be conducted on impacted canine treatment with clear aligner systems using clinical trials, wider age and ethnic groups, and presented with control groups treated with fixed appliances.

## 5. Conclusions

This systematic review confirms the promising possibility of impacted canine treatment using an orthodontic aligner system. It is an alternative to the therapy conducted with fixed appliances and rising in popularity due to being more aesthetically pleasing and providing greater comfort during treatment, but it has limitations. Additional auxiliaries like TADs, sectional wires, or elastics are still needed for the initial traction of the impacted canine. Only less severe malocclusions with cuspids positioned straight and directly in the alveolar ridge in young patients can be solely treated with clear aligners. Aligners allow for multiple treatment solutions, such as the canine-first approach, space creation for the canine in the arch, final canine traction, final aligning, and torque control. Due to the lower rigidity of some existing aligners compared to the orthodontic wire, higher periodontal ligament stress on the adjacent teeth can occur during aligner treatment, which may result in displacement of the anchorage teeth. A possible reduction in the overall treatment time is another aspect indicating why implementing aligner treatment into impacted canine malocclusion correction should be considered. To form a reliable and efficient treatment plan, more studies must be conducted with bigger sample sizes and fixed appliance control groups.

## Figures and Tables

**Table 1:** The Population, Intervention, Comparison, Outcomes, and Study Design (PICOS) framework.

	Inclusion Criteria	Exclusion Criteria
Population	Patients with impacted or significantly ectopic canines	-
Intervention	Orthodontic treatment carried out using aligner orthodontic therapy	Orthodontic treatment carried out without orthodontic aligners
Comparison	No comparison group was included	-
Outcomes	Any	Only tooth alignment after treatment
Study design	Any	Reviews, letters, editorials, commentaries, conference abstracts, books

**Table 2:** Search criteria.

Database	Search String
PubMed	(“Orthodontic aligner treatment” [All Fields] OR “Invisalign” [All Fields] OR “Clear aligner treatment”) AND (“Impacted Canine” [All Fields] OR “Impacted Tooth” [All Fields] OR “Ectopic Canine” [All Fields] OR “Ectopic Tooth” [All Fields])
Google Scholar	“Orthodontic aligner treatment” OR “Invisalign” OR “ Clear aligner treatment” AND “Impacted Canine” OR “Impacted Tooth” OR “Ectopic Canine” OR “Ectopic Tooth”
EBSCO Complementary Index MEDLINE Ultimate Springer Nature Journals Oxford Medicine Online Academic Search Ultimate Dentistry and Oral Sciences Source Elsevier Web of Science	TS = (“Orthodontic aligner treatment” OR “Invisalign” OR “Clear aligner treatment”) AND TS = (“Impacted Canine” OR “Impacted Tooth” OR “Ectopic Canine” OR “Ectopic Tooth”)

**Table 3:** General characteristics of studies.

Study	Group Size and Study Design	Patient Age	Patient Gender	Malposition of Canine	Period of Orthodontic Aligners Usage
Aldo Giancotti et al. 2021 [21]	1; case study	13	Female	High vestibular ectopic transposition with lateral displacement and low exposition	Whole treatment
Qijan Xia et al. 2022 [27]	1; 1 model was made into 4 3D finite models for 2 scenarios, 3D finite element analysis (FEA)	23	Male	Impacted in vestibular position, above premolar (scenario A) or above lateral incisor (scenario B)	Whole treatment
Mario Greco et al. 2022 [22]	2; different cases with different sequences of the treatment	16 and 43	Male and female	Impacted palatally	In cases 1 and 2, whole treatment except for hybrid treatment during canine traction with TAD
Gianluca Mampieri et al. 2021 [23]	1; case report	17	Female	Two slightly impacted in the alveolar crest	Whole treatment
Capuozzo R et al. 2023 [24]	2; case 1 had palatally displaced canine and case 2 had buccally displaced canine	17 and 18	Female	Case 1: impacted palatally Case 2: highly vestibular	After short period of traction with TAD
Memè L et al. 2024 [25]	1; case study	13	Female	Ectopic palatal with very low exposition	Whole treatment
Bocchino T et al. 2023 [26]	1; case report	19	Male	Impacted palatally	2nd phase of the treatment

**Table 4:** Detailed characteristics of studies.

Study	Treatment Protocol	TAD Use During Treatment	Usage of Fixed Orthodontic Appliance	Elastics Used	Aligner/Treatment Time
Aldo Giancotti et al. 2021 [21]	Resolve malocclusion and traction of ectopic canine into the arch, with the extraction of deciduous canine and two supernumeraries. Phase 1: Four months to create space for canines, with aligner changes every 2 weeks, then 5 months of canine traction with elastics. Phase 2: Residual canine extrusion and torque control, leveling the gingival anterior contour, with aligner changes every week. Phase 3: Class II correction with elastics and finishing.	No	No	Light auxiliary elastic stretched over the aligner between the palatal and buccal surfaces of the canine, then class II elastics to correct class II malocclusion and canine position.	24 months of treatment: Phase 1: 40 aligners, 9 months; Phase 2: 27 aligners, 3 months; Phase 3: 8 aligners, 12 months; Total: 67 aligners.
Qijan Xia et al. 2022 [27]	Finite element models were created from a male model, including the maxillary dentition with maxilla, periodontal ligaments, clear aligners, traction attachments, and right labial canine. Two scenarios were investigated: impacted canine above the premolar vestibular (A) and impacted canine impacted above the lateral incisor vestibular (B). Three traction models with clear aligners and one fixed appliance model were created for every scenario. Traction was performed with 0.6N elastics from the canine to the attached auxiliary (7 mm power arm, 3D attachment, or Angel Button on aligners and hook on fixed appliance). ABAQUS software (version 6.14; SIMULIA, Aix-en-Provence, France) was used for nonlinear iterative calculations.	No	Control scenario: 0.018 × 0.025 stiff wire.	Straight to the auxiliary (power arm, button, or 3D-printed “hook like” attachment).	Due to the characteristics of the FEA study, neither the treatment time nor number of aligners were the subject of the calculations.
Mario Greco et al. 2022 [22]	Case 1: Phase 1: Correcting malocclusion and recreating space for the impacted canine with aligners. Phase 2: Surgical exposure and canine disimpaction and traction using TADs and sectional wires. Phase 3: Refinement of canine position and finishing. Case 2: Phase 1: Canine-first technique with surgical exposure, disimpaction of canine with TADs and sectional wires, and displacement of the TAD during treatment. Phase 2: Final correction of canine position and torque with aligners, and finalization of wire extrusion.	Case 1: Palatally between premolars and the premolar and molar. Case 2: Mesially from 1st molar in the palate later in the vestibule between canine and 1st premolar.	Case 1: During canine traction, a sectional 0.18SS Australian wire was used from the canines to the TADs. Then, to not reposition them, a 0.17 × 0.25 TMA was used from the 2.5 to the canine labially. Case 2: During canine traction, a 150 g closed coil spring with a metal ligature was used from the canine to the TAD. For finishing, a sectional 0.016 × 0.022 CuNiTi wire from the lateral to the 1st premolar was used.	Triangular criss-cross.	Case 1: 18 months of treatment: Phase 1: 25 aligners; Phase 2: TAD and sectional wire; Phase 3: 18 aligners; Total: 43 aligners. Case 2: Approximately 24 months of treatment: Phase 1: TAD + sectional wire; Phase 2: 38 aligners + sectional wire; Total: 38 aligners.
Gianluca Mampieri et al. 2021 [23]	Phase 1 was aimed at recovering space in the arch for canines, expansion, and the correction of incisor inclination. Phase 2 included deciduous canine removal, surgical exposure of both canines, and cuspid traction with elastic forces from the canines to the aligner and eruption guides. Phase 3 included residual positioning of canines and finishing for better esthetic and occlusal outcomes.	No	No	Intra-arch 6 mm/180 g elastics from canine hooks to buttons hidden inside pontics over the aligners, then replaced with buttons on canines, dovetail hook on aligners and 4 mm/180 g elastics.	18 months of treatment: Phase 1: 21 aligners; Phase 2: 17 aligners; Phase 3: 13 aligners; Total: 61 aligners.
Capuozzo R et al. 2023 [24]	Case 1: Phase 1: Surgical exposure of canines, traction of impacted canines using TAD and sectional wire in cantilever spring (canine-first technique), and then extraction of the deciduous one. Phase 2: Use of aligners with bite ramps for leveling and alignment of both arches, and traction of canines with elastics. Phase 3: Residual canine traction and torque, followed by finishing. Case 2: Phase 1: Surgical exposure of canines, extraction of deciduous canines, and traction using TAD with cantilever spring (canine-first technique). Phase 2: Aligners used for space creation with eruption compensator, alignment, and leveling of both arches; class II elastics used to improve occlusion and canine traction. Phase 3: Aligners used for expansion to resolve crowding and transverse discrepancy, followed by torquing and final positioning of the canine.	Case 1: Palatally between 1.5 and 1.6. Case 2: Between 2.5 and 2.6 buccally.	Case 1: A 0.017 x 0.025 TMA 50 g cantilever spring was used from the canine to the TAD. Case 2: A 0.018 × 0.025 TMA 50 g cantilever spring from the TAD to the canine was used.	Case 1: Elastic ligature from the canine to the eruption compensator. Case 2: From the canine to 3.6, class II elastics and later, wrap around aligner elastics to a buccal button on the canine.	Case 1: 20 months of treatment: Phase 1: TAD + sectional wire; Phases 2 and 3: Approx 52 aligners (14 months). Case 2: 18 months of treatment: Phase 1: TAD + sectional wire; Phases 2 and 3: Approx 48 aligners (12 months).
Memè L et al. 2024 [25]	Phase 1: Upper arch expansion to make space for canines with pontics. Phase 2: Increase in posterior vertical dimension through fake build-ups implemented in aligners, followed by canine traction. Phase 3: Occlusion refinement, short class II for better canine position, and mesial derotation of molars.	No	No	Class II elastics with a size of 3/16” and 4.5 ounces were used from the canines to the lower premolars.	21 months of treatment: Phase 1: 7 aligners; Phase 2: 31 aligners; Phase 3: 26 aligners; Total: 64 aligners.
Bocchino T et al. 2023 [26]	Phase 1: Canine-first approach; this procedure included surgical disimpaction of the canine, moving the crown away from the roots of the adjacent incisors with a cantilever and a skeletal anchorage (TAD). Phase 2: Canine traction with Alastik to 5.3 and composite ramping. Phase 3: Extraction of 5.3, sectional brackets on 1.6 and 1.3 with a cantilever for canine traction and torque. Phase 4: Alignment and mesialization of posterior segments and the canine into absent lateral incisor with aligners, followed by class III elastics to promote desired movements.	Between 2nd premolar and 1st molar palatally.	A 0.019 x 0.025 TMA 100 g cantilever spring from the TAD to the canine was used, with a bracket on 1.6 with the TAD for additional anchorage. A 0.019 × 0.025 TMA cantilever was used on the canine vestibular, followed by a bracket on the canine.	Alastik from 53 to 13 during the second course of traction, followed by class III on the canines during aligner treatment.	14 months + phase 3 treatment time; Phase 1: TAD + sectional wire (3 months); Phase 2: Alastik from 53 to 13 (4 months); Phase 3: Sectional wire bonded on 16 and 13 + TAD; Phase 4: 24 aligners (7 months).

**Table 5:** Risk of bias evaluation of the included studies.

Checklist Question	Aldo Giancotti et al. [21]	Mario Greco et al. [22]	Gianluca Mampieri et al. [23]	Capuozzo R et al. [24]	Memè L et al. [25]	Bocchino T et al. [26]
Were the patient’s demographic characteristics clearly described?	Yes	Yes	Yes	Yes	Yes	Yes
Was the patient’s history clearly described and presented as a timeline?	Yes	Yes	Yes	Yes	Yes	Yes
Was the current clinical condition of the patient upon presentation clearly described?	Yes	Yes	Yes	Yes	Yes	Yes
Were diagnostic tests or assessment methods and the results clearly described?	Yes	Yes	Yes	Yes	Yes	Yes
Was the intervention(s) or treatment procedure(s) clearly described?	Yes	Yes	Yes	Yes	Yes	Yes
Was the post-intervention clinical condition clearly described?	Yes	Yes	Yes	Yes	Yes	Yes
Were adverse events (harms) or unanticipated events identified and described?	No	No	No	No	No	No
Does the case report provide takeaway lessons?	Yes	Yes	Yes	Yes	Yes	Yes

## Data Availability

Data supporting the findings of this study are available within the article.
